# Robust optimization of VMAT for prostate cancer accounting for geometric uncertainty

**DOI:** 10.1002/acm2.13738

**Published:** 2022-08-03

**Authors:** Takuya Wada, Daisuke Kawahara, Yuji Murakami, Takeo Nakashima, Yasushi Nagata

**Affiliations:** ^1^ Section of Radiation Therapy Department of Clinical Practice and Support Hiroshima University Hospital Minami‐ku Japan; ^2^ Department of Radiation Oncology Institute of Biomedical and Health Sciences Hiroshima University Hospital Minami‐ku Japan

**Keywords:** prostate, RayStation, robust optimization, uncertainty

## Abstract

The aim of this study was to propose optimal robust planning by comparing the robustness with setup error with the robustness of a conventional planning target volume (PTV)‐based plan and to compare the robust plan to the PTV‐based plan for the target and organ at risk (OAR). Data from 13 patients with intermediate‐to‐high‐risk localized prostate cancer who did not have T3b disease were analyzed. The dose distribution under multiple setup error scenarios was assessed using a conventional PTV‐based plan. The clinical target volume (CTV) and OAR dose in moving coordinates were used for the dose constraint with the robust plan. The hybrid robust plan added the dose constraint of the PTV‐rectum to the static coordinate system. When the isocenter was shifted by 10 mm in the superior–inferior direction and 8 mm in the right‐left and anterior directions, the doses to the CTV, bladder, and rectum of the PTV‐based plan, robust plan, and hybrid robust plan were compared. For the CTV D_99%_ in the PTV‐based plan and hybrid robust plan, over 95% of the prescribed dose was secured in all directions, except in the inferior direction. There was no significant difference between the PTV‐based plan and the hybrid robust plan for rectum V_70Gy_, V_60Gy_, and V_40Gy_. This study proposed an optimization method for patients with prostate cancer. When the setup error occurred within the PTV margin, the dose robustness of the CTV for the hybrid robust plan was higher than that of the PTV‐based plan, while maintaining the equivalent OAR dose.

## 1 INTRODUCTION

The volume required for target tumors is determined by the radiation therapy plan.^1^, ^2^ The clinical and anatomical concepts of tumors define the gross tumor volume and clinical target volume (CTV). To administer a sufficient dose to the CTV, a margin must be added. Internal margin (IM) is a range that includes uncertainty due to physiological variations, such as respiratory motion, in the planning. The setup margin (SM) is a range that accounts for uncertainty in patient positioning. The planning target volume (PTV) is defined by taking into account IM and SM to achieve the clinical goal.^3^, ^4^ Prescriptions to the PTV rely upon a geometric concept in static coordinates, not dose prescriptions to a true target in moving coordinates. The dose distribution, not the geometric margin in the moving coordinates, determines whether a CTV receives a prescribed dose.^5^, ^6^

In heavy ion and proton therapy, the main idea is to have a robust plan.^5^, ^7^, ^8^ Because the dose distribution changes significantly due to the use of the Bragg peak and slight changes in density in proton radiotherapy, it is vulnerable to position fluctuations. Because the dose distribution in heavy ion and proton radiotherapy can fluctuate significantly, the idea of ​​PTV is not effective.^9^ Thus, rather than the PTV margin, dose robustness should be discussed in terms of dose distribution. This robust planning technique has not been widely used in photon radiotherapy. At the research level, there are some reports on the robust planning of photon radiotherapy.^10^, ^11^, ^12^, ^13^ When compared to conventional radiotherapy, a robust plan for volumetric modulated arc therapy (VMAT) in the lung secured the tumor dose while reducing the dose to normal tissues.^9^ Mahmoudzadeh et al. showed that a robust plan with intensity‐modulated radiotherapy (IMRT) reduced the dose to the heart when compared to the dose for breast cancer using conventional IMRT.^11^ For glioblastoma, the target dose was equivalent to that of conventional IMRT, and the cerebral cortex was better preserved than in conventional IMRT.^14^ These studies evaluated the robust optimization for a target that was not near the organ at risk (OAR). For prostate cancer, even if a setup error occurs, the target dose is sufficient, and the bladder and rectum suppress the dose as much as possible. In the clinical setting, daily cone beam computed tomography analysis has revealed a decrease in the dose of CTV, which has been linked to an increase in the number of prostate cancer recurrences.^15^ Jin et al. reported that the assessment of the dose uncertainty in prostate IMRT leads to tumor control and risk reduction, which requires treatment planning that takes dose uncertainty into account.^16^ Recently, a novel function was developed in RayStation (RaySearch Medical Laboratories AB, Stockholm, Sweden). It is capable of incorporating uncertainty into the planning optimization process and generating a robust plan.^17^ The robust plan has the advantage of identifying the best scenario for the trade‐off between dose robustness and OAR constraints.^18^ However, the dose constraint for the robust plan is yet to be determined. Conventionally, the beam arrangement and optimization were performed in such a way that the prescription dose in the static coordinates (PTV‐based plan) was delivered to the PTV.^19^ Therefore, between PTV‐based and robust plans, the concept of optimization differs. The optimal dose constraint should be determined for a robust plan. The current study focused on the prostate adjacent to the OARs. This study aimed to determine the treatment plan method with a robust plan by evaluating the robustness of a moving target with a PTV‐based plan. Moreover, the robust and conventional PTV‐based plans for the target and OARs were compared.

## 2 MATERIALS AND METHODS

### 2.1 Patient characteristics

This study included patients with localized prostate cancer who underwent VMAT at university hospital. The researchers used data from 13 patients with intermediate‐to‐high‐risk localized prostate cancer without T3b disease. In our hospital, patients with intermediate‐to‐high‐risk prostate cancer are treated with 78 Gy in 39 fractions. By contrast, patients with low‐risk prostate cancer are treated with 74 Gy in 37 fractions. Thus, the patients with intermediate‐to‐high‐risk prostate cancer were selected for the current study. The National Comprehensive Cancer Network guidelines for prostate cancer were used to determine the risk classification.

### 2.2 PTV‐based plan

The planning CT scans were conducted with a slice thickness of 2.5 mm on LightSpeed16 (GE Healthcare, Chicago, IL, USA). RayStation Ver.6 was used for contouring, treatment planning, and dose evaluation. A region of interest of the whole prostate was generated using magnetic resonance imaging. Radiation oncologists used our Institution's protocol to create the contouring CTV, which was used in clinical patients. CTV was defined as the prostate and half seminal vesicles. The PTV margin was added to the CTV — 10 mm to the superior and inferior, 8 mm to the anterior, right, and left, and 6 mm to the posterior. The target for the prescription was the PTV minus the part that overlapped with the rectum. The rectum and bladder were described as the OARs. The region of the rectum was defined as a 4‐mm rectal wall. The region of the rectum overlap was defined as the area where the entire rectum and PTV overlapped. The bladder was defined as the 4‐mm bladder wall. A total of 78 Gy in 39 fractions were prescribed for the PTV‐rectum. The dose constraints used in the optimization parameters are listed in the appendix. A TrueBeam linear accelerator (Varian Medical Systems, CA, USA) was used in this study. A photon beam energy of 10 MV was chosen for this study. The VMAT plan was created with a partial arc of 210–150°. The collimator angle was set to 10°. The center of the CTV was defined as the isocenter. Table S1 summarizes the optimization parameters.

### 2.3 Robust plan

**FIGURE 1:**
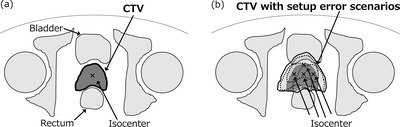
Illustrations of clinical target volume (CTV) in the planning target volume (PTV)‐based plan (a) and CTV in the robust optimization planning (b)

A robust plan was generated from the dose distribution of multiple setup error scenarios using RayStation Ver.6. The robust optimization aims at minimizing the expected value of the worst‐case distribution.^17^ The number of scenarios scales up in proportion to the degree of uncertainty and the number of shift directions. These scenarios include a no setup error scenario, endpoint setup error scenario, and intermediate setup error scenario in the right‐left, inferior‐superior, and posterior‐anterior directions. The setup error, which was assumed to occur within the PTV margin, was determined to be the scenario.^20^ Namely, the CTV dose with the setup error in the PTV‐based plan, which was used for the prescribed dose for the robust plan, was evaluated. The dose distribution in the moving coordinates of the PTV‐based plan determined the dose constraint in the robust plan. For the robust plan in the moving coordinates system, the robust optimization function was used for CTV. Figure 1 shows illustrations of CTV in the PTV‐based plan and CTV in the robust optimization planning. The robust plan optimization parameters are listed in the appendix. The robust optimization function of the robust plan was used only for the CTV. The optimization parameters of the CTV and OAR in the robust plan were determined by the dose of CTV and OAR when the PTV‐based plan was shifted by 8 mm in the right, left, and anterior directions and 10 mm in the inferior and superior directions. The median values of D_1%_ and D_99%_ of the CTV in all directions were used for target optimization in the robust plan. The median values in all patients with average V_75Gy_, V_70Gy_, V_60Gy_, and V_40Gy_ of the rectum, and V_75Gy_, V_70Gy_, V_60Gy_, and V_40Gy_ of the bladder were used as optimization parameters in the robust plan for the OAR. The optimization parameters are summarized in Table S2. The dose constraints of robust optimization were established to be equivalent to the rectum and bladder dose of the PTV‐based plan. We recalculated the PTV‐based plan with multiple setup error scenarios and determined the rectal and bladder dose constraints from the calculated dose‐volume histogram. In this study, we did not intentionally normalize the robust plan with PTV. The common normalization was not suitable because the PTV‐based plan was delivered directly to PTV, and the robust plan using robust optimization algorithms was based on CTV.^9^

### 2.4 Hybrid robust plan

The hybrid robust plan was generated using RayStation. For the target optimization parameters, the robust plan used the CTV in the moving coordinates, and the PTV‐based plan used the PTV in the static coordinates. The hybrid robust plan was defined as the one that utilized both the PTV in the static coordinates and the CTV in the moving coordinates. As shown in Table S3, the optimization parameters of the PTV‐rectum used in the PTV‐based plan were added to the optimization parameters and setup error scenarios of the robust plan.

### 2.5 Plan evaluation

For the PTV‐based plan, robust plan, and hybrid robust plan, the isocenter was shifted by 10 mm in the superior‐inferior direction and 8 mm in the right‐left and anterior directions. The range of the shifted isocenter was within the PTV margin. In the moving coordinate system, the isocenter is moved to three axes in the three axes defined as lateral (right–left), longitudinal (superior–inferior), and vertical (anterior–posterior). The target and OAR dose were evaluated with the dose distribution calculated with the moving coordinate. For each patient, the doses of the CTV, rectal wall, and bladder wall were calculated. The rectal and bladder walls were both 4‐mm thick. The points of evaluation were D_99%_ and D_1%_ of the CTV, V_75Gy_, and V_70Gy_, V_60Gy_, V_40Gy_ of the rectum, V_75Gy_ and V_70Gy_, V_60Gy_, V_40Gy_, of the bladder.^21^, ^22^, ^23^

### 2.6 Statistics

A *t*‐test was used to compare the difference between the hybrid robust plan and the PTV‐based plan or the robust plan in terms of dose robustness. The statistical significance threshold was set at *p* < 0.01 to be considered statically significant.

## 3 RESULTS

**FIGURE 2:**
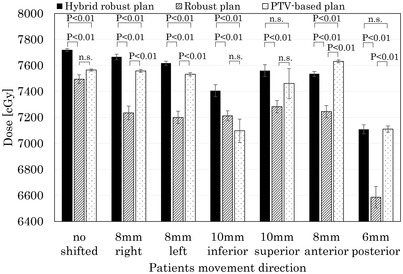
Comparison of clinical target volume (CTV) D_99%_ between the hybrid robust plan, the robust plan, and the planning target volume (PTV)‐based plan

Figure 2 shows a comparison of the D_99%_ of the CTV between the PTV‐based robust plan and the hybrid robust plan when the isocenter shifted within the PTV margin. For the robust and the hybrid robust plans, the prescription was normalized at the mean dose of the CTV (CTV Dmean) for each plan, with setup error in the PTV‐based plan. The CTV Dmean ± 2SD of the robust plan, the hybrid robust plan, and the PTV‐based plan were 7930 ± 131 cGy, 7962 ± 75 cGy, and 7866 ± 69 cGy. The hybrid robust and the robust plans had a significantly higher CTV Dmean than the PTV‐based plan. There was no significant difference in CTV Dmean between the hybrid robust plan and the robust plan. In the CTV D_99%_, the hybrid robust plan was significantly higher than the robust plan in all directions and significantly higher than the PTV‐based plan except in superior and posterior directions. The robust plan was not significantly higher than the PTV‐based plan in CTV D_99%_. On the posterior side, the PTV margin was lower to reduce the rectal dose, and we only provided reference information. Except for the inferior direction, over 95% of the prescribed dose was secured in all directions in the PTV‐based plan. The mean dose of CTV D_99%_ in the inferior direction was 7097 cGy, which was less than the prescribed dose of approximately 9%. In the hybrid robust plan, over 95% of the prescribed dose was secured in all shift directions. When the isocenter shifted within the PTV margin and the CTV D_99%_ was compared between the hybrid robust plan and the robust plan, the robust plan had less than 95% of the prescribed dose in all directions. The robust plan removed the PTV‐rectum from dose constraints. Obviously, in the hybrid robust plan that included the PTV‐rectum in dose constraints, the CTV D_99%_ was higher. The CTV dose tends to be significantly reduced when the robust plan has a setup error.

**FIGURE 3:**
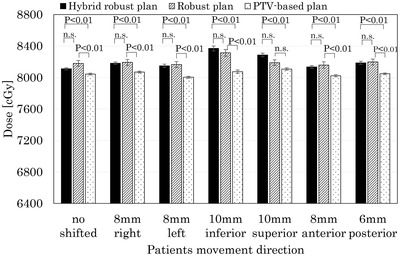
Comparison of clinical target volume (CTV) D1% between the hybrid robust plan, the robust plan, and the planning target volume (PTV)‐based plan

Figure 3 shows a comparison of the D_1%_ of the CTV between the PTV‐based, robust, and hybrid robust plans when the isocenter shifted direction. Across the board, D_1%_ of CTV in the hybrid robust plan was significantly higher than in the PTV‐based plan. The robust plan was significantly higher than the PTV‐based plan except in the superior direction. There was no significant difference between the hybrid robust plan and the robust plan. When the hybrid robust plan shifted 10 mm in the inferior direction, the D_1%_ of CTV was the highest at 8378 cGy.

**FIGURE 4:**
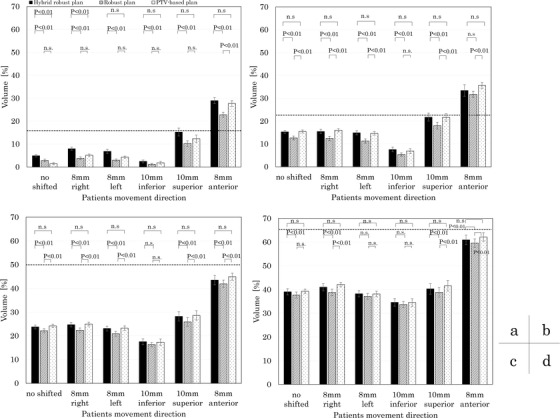
Comparison of rectum dose between the hybrid robust plan, the robust plan, and the planning target volume (PTV)‐based plan. (a) V_75Gy_, (b)V_70Gy_, (c)V_65Gy_, and (d)V_40Gy_

The comparison of the mean values and standard errors of the rectum V_75Gy_, V_70Gy_, V_60Gy_, and V_40Gy_ for the PTV‐based plan, robust plan, and hybrid robust plan is shown in Figure 4. The plotted dash line for each metric indicates the clinically acceptable level at no setup error scenarios.^21^, ^22^, ^23^ The rectal dose increased when there were setup errors in the anterior and superior directions. The hybrid robust plan has a significantly higher dose than the PTV‐based plan for the V_75Gy_ of the rectum with no setup error and 8 mm to the right. There was no significant difference between the PTV‐based plan and the hybrid robust plan for rectum V_70Gy_, V_60Gy_, and V_40Gy_. The rectal dose in all metrics of the robust plan did not have a significantly higher dose than the PTV‐based plan.

**FIGURE 5:**
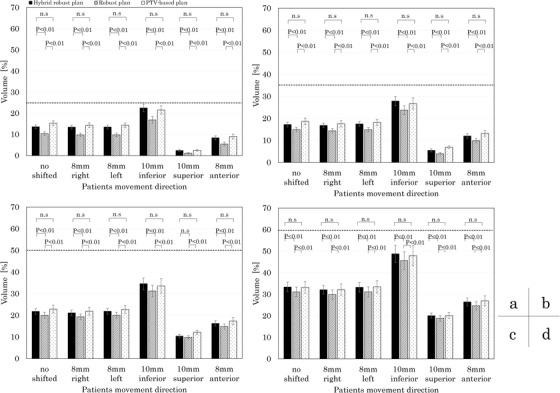
Comparison of bladder dose between the hybrid robust plan, the robust plan, and the planning target volume (PTV)‐based plan. (a) V_75Gy_, (b)V_70Gy_, (c)V_65Gy_, and (d)V_40Gy_

The comparison of the mean values and standard errors of the bladder V_75Gy_, V_70Gy_, V_60Gy_, and V_40Gy_ between the PTV‐based plan, robust plan, and hybrid robust plan is shown in Figure 5. The bladder dose increased when there was a setup error in the inferior direction. There was no significant difference between the PTV‐based plan and the hybrid robust plan for bladder V_75Gy_, V_70Gy_, V_60Gy_, and V_40Gy_. The robust plan was significantly lower than those in the hybrid robust plan and the PTV‐based plan in V_75Gy_, V_70Gy_, V_60Gy_, and V_40Gy_.

## 4 DISCUSSION

For conventional treatment planning assessment, PTV coverage in static coordinates was regarded as important. However, the concept of PTV has limitations.^5^ The target received the ideal dose distribution without taking into account the OAR dose because the PTV was assumed to be not equally conformal on all sides of the CTV. In other words, the dose distribution is assumed to be completely homogeneous within the PTV and to have a constant spread in all directions outside of it. The prostate is not spherical in clinical radiotherapy for prostate cancer, and it is adjacent to risk organs like the bladder and rectum. To reduce the exposure to OAR as much as possible, the dose distribution with VMAT does not have a uniform spread outside the PTV. In this study, we used clinical patients and used the shifting isocenter to evaluate the dose of the target and OAR in the moving coordinates. At the prescribed dose, the PTV‐based plan showed a dose reduction of approximately 9%. The difference in the geometrical shape concept and the real shape of the prostate of a patient being administered a dose for prostate cancer affects the extent of dose reduction. The CTV dose was significantly reduced in the inferior direction even when the isocenter position was shifted within the PTV margin. For the hybrid robust plan, a dose of 95% or more of the prescribed dose was delivered to the CTV. This indicates that the hybrid robust plan is maintained at a higher dose than the PTV‐based plan. The hybrid robust plan has higher maximum and rectal doses than the PTV‐based plan. However, for RTOG 0126 and other reports, the rectal dose with the hybrid robust plan met the dose constraint of the dose standard.^23^, ^24^ Robust optimization can optimize one target with setup error within one scenario. Namely, the uneven expansion of the CTV in the direction of the respective organs of the rectum and bladder cannot be considered. Future research will determine if robust optimization improves multiple scenarios.

The clinical outcomes of the PTV‐based plan were promising, and robust hybrid plans were designed to keep the PTV‐based plan. The goal of this research was to improve dose robustness by reducing the worst‐case scenarios such as a significantly lower target dose or a significantly higher dose of the OAR for the PTV‐based plan. The PTV‐based and robust plans have different prescription targets and optimization methods. Therefore, they were not normalized on purpose. Liang et al. study reported that both the PTV‐based and robust optimization cannot be the same, and the traditional clinical prescription for the robust plan is inappropriate.^9^ Therefore, in this study, for the dose constraints of CTV and OAR in the robust plan, we used conventional clinical data. When setup errors occurred, the doses of CTV and OAR were calculated. Moreover, the dose constraint of the PTV‐rectum supports the robustness of the CTV dose. In the hybrid robust plan, the PTV‐rectum was required to ensure coverage of the CTV dose, despite variations in the setup position. The hybrid robust plan helped to avoid dose reduction in the robust plan. The robust optimization in RayStation is based on minimax optimization, which considers the optimization functions that are robust in a worst‐case scenario.^17^ With a similar number of scenarios as in the current study, Byrne et al. demonstrated that a robust plan is resistant to setup errors.^10^ The proposed hybrid robust method improves the target coverage while maintaining the nonsignificant difference in OAR dose in most of the random setup errors as compared with the conventional robust plan. From above, the proposed dose constraints and scaling of the prescription dose can be proposed as a practical method for determining the dose constraint of robust optimization.

## 5 CONCLUSIONS

This study proposes a robust optimization method for prostate VMAT. The dose robustness of the CTV when the setup error occurred within the PTV margin was higher for the hybrid robust plan than for the PTV‐based plan while maintaining equivalent OAR dose. The current study suggests that a robust optimization can reduce dose uncertainty due to setup errors.

## CONFLICT OF INTEREST

The authors declare that there is no conflict of interest that could be perceived as prejudicing the impartiality of the research reported.

## AUTHOR CONTRIBUTIONS

TW and DK conceived and designed the study, and write the manuscript. TW performed data collection, data analysis and interpretation of results. WT, DK, YM, TN and YN helped interpret the data and write the manuscript.

## Supporting information

Supporting Information

Supporting Information

## Data Availability

The data that support the findings of this study are available from the corresponding author upon reasonable request.
