# Supplementary material for: Robust optimization of VMAT for prostate cancer accounting for geometric uncertainty
Source: J Appl Clin Med Phys. 2022 Aug 3;23(9):e13738. doi: 10.1002/acm2.13738 (PMC9512334; doi:10.1002/acm2.13738)
Supplement: Supplementary file 1 — Supporting Information [file ACM2-23-e13738-s001.docx]

**APPENDIX A**

OPTIMIZATION PARAMETRS

**Table S1.** The optimization parameters of PTV-based plan

| ROI | Type | Dose[cGy] | Target | Volume[%] |
| --- | --- | --- | --- | --- |
| PTV-rectum | Max dose | 8200 |  |  |
| PTV-rectum | Uniform dose | 7900 |  |  |
| PTV-rectum | Min DVH |  | 7800 | 100 |
|  |  |  |  |  |
| Rectum | Max DVH |  | 4000 | 40 |
| Rectum | Max DVH |  | 6000 | 20 |
| Rectum | Max DVH |  | 7000 | 15 |
| Rectum | Max DVH |  | 7800 | 0 |
|  |  |  |  |  |
| Rectum overlap | Max dose | 7350 |  |  |
| Rectum overlap | Min dose | 7000 |  |  |
|  |  |  |  |  |
| Bladder | Max DVH |  | 4000 | 50 |
| Bladder | Max DVH |  | 6000 | 35 |
| Bladder | Max DVH |  | 7000 | 25 |

**Table S2.** The optimization parameters of the robust plan

| ROI | Type | Dose[cGy] | Target | Volume[%] |
| --- | --- | --- | --- | --- |
| rCTV | Max dose | 8045 |  |  |
| rCTV | Min DVH |  | 7553 | 100 |
|  |  |  |  |  |
| Rectum | Max DVH |  | 4000 | 40 |
| Rectum | Max DVH |  | 6000 | 24 |
| Rectum | Max DVH |  | 6500 | 20 |
| Rectum | Max DVH |  | 7000 | 16 |
| Rectum | Max DVH |  | 7500 | 4 |
| Rectum | Max DVH |  | 7800 | 0 |
|  |  |  |  |  |
| Rectum overlap | Max dose | 7350 |  |  |
| Rectum overlap | Min dose | 7000 |  |  |
|  |  |  |  |  |
| Bladder | Max DVH |  | 4000 | 29 |
| Bladder | Max DVH |  | 6000 | 20 |
| Bladder | Max DVH |  | 6500 | 18 |
| Bladder | Max DVH |  | 7000 | 16 |
| Bladder | Max DVH |  | 7500 | 13 |
| Bladder | Max DVH |  | 8000 | 0 |

**Table S3.** The optimization parameters of the hybrid robust plan

| ROI | Type | Dose[cGy] | Target | Volume[%] |
| --- | --- | --- | --- | --- |
| PTV-rectum | Mac dose | 8200 |  |  |
| PTV-rectum | Uniform dose | 7900 |  |  |
| PTV-rectum | Min DVH |  | 7800 | 100 |
|  |  |  |  |  |
| rCTV | Max dose | 8045 |  |  |
| rCTV | Min DVH |  | 7553 | 100 |
|  |  |  |  |  |
| Rectum | Max DVH |  | 4000 | 40 |
| Rectum | Max DVH |  | 6000 | 24 |
| Rectum | Max DVH |  | 6500 | 20 |
| Rectum | Max DVH |  | 7000 | 16 |
| Rectum | Max DVH |  | 7500 | 4 |
| Rectum | Max DVH |  | 7800 | 0 |
|  |  |  |  |  |
| Rectum overlap | Max dose | 7350 |  |  |
| Rectum overlap | Min dose | 7000 |  |  |
|  |  |  |  |  |
| Bladder | Max DVH |  | 4000 | 29 |
| Bladder | Max DVH |  | 6000 | 20 |
| Bladder | Max DVH |  | 6500 | 18 |
| Bladder | Max DVH |  | 7000 | 16 |
| Bladder | Max DVH |  | 7500 | 13 |
| Bladder | Max DVH |  | 8000 | 0 |
