# Supplementary material for: Robust optimization of VMAT for prostate cancer accounting for geometric uncertainty
Source: J Appl Clin Med Phys. 2022 Aug 3;23(9):e13738. doi: 10.1002/acm2.13738 (PMC9512334; doi:10.1002/acm2.13738)
Supplement: Supplementary file 2 — Supporting Information [file ACM2-23-e13738-s002.docx]

Title: Robust optimization of VMAT for prostate cancer accounting for geometric uncertainty

**Author name**

Autors: Takuya Wada^a^, Daisuke Kawahara^b^, Yuji Murakami^b^, Takeo Nakashima^a^, and

Yasushi Nagata^b^

a: Section of Radiation Therapy, Department of Clinical Practice and Support, Hiroshima University Hospital, Kasumi 1-2-3, Minami-ku, Hiroshima, 734-8551, Japan

b: Department of Radiation Oncology, Institute of Biomedical and Health Sciences, Hiroshima University Hospital, Kasumi 1-2-3, Minami-ku, Hiroshima, 734-8551, Japan

**Affiliations**

**Takuya Wada (First Author)**,

Section of Radiation Therapy, Department of Clinical Practice and Support,

Hiroshima University Hospital,

Kasumi 1-2-3, Minami-ku, Hiroshima City,

Hiroshima, Japan 734-8551

wat@hiroshima-u.ac.jp

Substantial contributions to the conception or design of the work and the acquisition, analysis, interpretation of data for the work.

**Daisuke Kawahara (Second Author, Corresponding Author)**,

Department of Radiation Oncology, Institute of Biomedical and Health Sciences,

Hiroshima University Hospital,

Kasumi 1-2-3, Minami-ku, Hiroshima City,

Hiroshima, Japan 734-8551

daika99@hiroshima-u.ac.jp

Substantial contributions to the conception or design of the work and the acquisition, analysis, interpretation of data for the work.

**Yuji Murakami (Third Author)**,

Department of Radiation Oncology, Institute of Biomedical and Health Sciences,

Hiroshima University Hospital,

Kasumi 1-2-3, Minami-ku, Hiroshima City,

Hiroshima, Japan 734-8551

yujimura@hiroshima-u.ac.jp

Drafting the work or revising it critically for important intellectual content

**Takeo Nakashima (Fourth Author)**,

Section of Radiation Therapy, Department of Clinical Practice and Support,

Hiroshima University Hospital,

Kasumi 1-2-3, Minami-ku, Hiroshima City,

Hiroshima, Japan 734-8551,

mla@hiroshima-u.ac.jp,

Drafting the work or revising it critically for important intellectual content

**Yasushi Nagata (Fifth Author)**,

Department of Radiation Oncology, Institute of Biomedical and Health Sciences,

Hiroshima University Hospital,

Kasumi 1-2-3, Minami-ku, Hiroshima City,

Hiroshima, Japan 734-8551

nagat@hiroshima-u.ac.jp

Final approval of the version to be published

**Running Tittle：** Robust optimization for prostate cancer

**Keywords：** prostate, robust optimization**,** uncertainty, RayStation
